# Dysfunction of RNA/RNA-Binding Proteins in ALS Astrocytes and Microglia

**DOI:** 10.3390/cells10113005

**Published:** 2021-11-03

**Authors:** Simona Rossi, Mauro Cozzolino

**Affiliations:** Istituto di Farmacologia Traslazionale, CNR, 00133 Rome, Italy

**Keywords:** Amyotrophic Lateral Sclerosis, neuroinflammation, RNA metabolism, RNA-binding proteins, astrocytes, microglia, FUS, TDP-43, C9orf72

## Abstract

Amyotrophic Lateral Sclerosis is a neurological disease that primarily affects motor neurons in the cortex, brainstem, and spinal cord. The process that leads to motor neuron degeneration is strongly influenced by non-motor neuronal events that occur in a variety of cell types. Among these, neuroinflammatory processes mediated by activated astrocytes and microglia play a relevant role. In recent years, it has become clear that dysregulation of essential steps of RNA metabolism, as a consequence of alterations in RNA-binding proteins (RBPs), is a central event in the degeneration of motor neurons. Yet, a causal link between dysfunctional RNA metabolism and the neuroinflammatory processes mediated by astrocytes and microglia in ALS has been poorly defined. In this review, we will discuss the available evidence showing that RBPs and associated RNA processing are affected in ALS astrocytes and microglia, and the possible mechanisms involved in these events.

## 1. Introduction

Amyotrophic Lateral Sclerosis (ALS) is a disease of motor neurons and neighbouring non-neuronal cells. ALS affects humans either as a sporadic, non-familial form or as a less frequent familial form, with an autosomal dominant inheritance pattern [1]. In both cases, several associated/causative gene mutations have been identified, and thus the study of genetic ALS is at the centre of intense research efforts in devising effective therapeutic strategies that are still lacking [2,3]. 

The vast majority of genetic ALS is attributable to mutations in the SOD1, TDP-43, FUS, and C9orf72 genes [4]. The mechanisms which account for ALS have been deeply investigated in the last 30 years, and impairment in protein quality control, alterations in mitochondrial function, cytoskeletal and axonal defects, and dysfunctional RNA metabolism have emerged as leading pathogenic processes. Yet, many other mechanisms have been shown to play a role, further testifying for the complexity and the multifactorial nature of the disease [5]. Studies on SOD1, which was the first gene to be identified in familial ALS [6], established the paradigm that ALS is a non-cell-autonomous disease, with non-motor neuronal cells, such as astrocytes, microglia, muscle cells, oligodendrocytes, and others, being equally affected by the disease mechanisms and having an active role in the overall disease process. In particular, neuroinflammatory events orchestrated by activated astrocytes and microglia cells occur in both sporadic and familial ALS and play a crucial role in disease progression [7,8]. More recently, a contribution of the immune response in ALS progression has been established, thus adding further complexity to the multi-systemic nature of this disease [9,10]. Studies on FUS, TDP-43, and C9orf72 have introduced the concept that alterations of several processes that regulate RNA metabolism have a key role in pathogenic events. Indeed, RNA transcription, splicing, transport, stability, and translation, are affected in ALS motor neurons by both dysfunctional mutant TDP-43 and FUS, two RNA-binding proteins (RBPs) with established roles in the control of RNA metabolism, as well as by GGGGCC (G4C2) hexanucleotide repeat expansions (HRE) in the first intron of the C9orf72 gene, which affect RNA processing by an RNA-mediated gain-of-function mechanism [11,12]. Whether the two paradigms are connected, i.e., whether there is an interplay between defects in RBP/RNA metabolism and neuroinflammation in ALS, is still poorly characterized. In this review, we want to provide an overview of what has been described so far about a possible role of reactive microglia and astrocytes in ALS linked to TDP-43, FUS, and C9orf72 mutations, and how dysfunctional RBP and associated RNA processing might be involved in these events.

## 2. Glia-Mediated Neuroinflammation in TDP-43, FUS, and C9orf72 ALS

Astrocytes and microglia are essential cell types in the central nervous system (CNS), performing a vast range of functions, which are complementary to each other, to maintain the proper homeostasis of the CNS. Astrocytes, which are the most abundant non-neuronal cells in the CNS, provide metabolic support for neurons, are involved in the maintenance of blood–brain barrier integrity, modulate neuronal activity through the secretion of cytokines, chemokines, and gliotransmitters, and by controlling the proper ionic and neurotransmitter homeostasis, including glutamate buffering [13]. Microglia are the resident immune-competent cells of CNS, and constantly control the local microenvironment thus rapidly reacting to any stimuli that perturb the CNS homeostasis, such as infections or neuronal injury [14]. Further, microglia exert immune-independent functions in the developing as well as in the adult brain, thus extending the functional relevance of this cell type. In neurodegenerative conditions such as those characterizing ALS, microglia fine-tune their gene expression profiles and assume a range of different reactive states, which can be associated with different phases of the disease [15,16]. Similarly, astrocytes become reactive in response to various stimuli, a process commonly referred to as astrogliosis: they change their morphology, proliferate, and secrete pro- or anti-inflammatory molecules [17,18,19].

**Figure 1:**
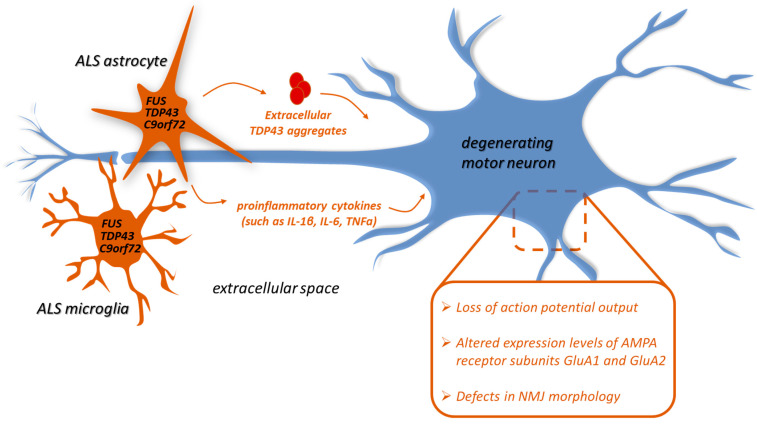
**Non**-**cell-autonomous mechanisms underlying motor neuron degeneration mediated by ALS-linked C9orf72, FUS, or TDP-43 astrocytes and microglia.** Wild-type motor neurons undergo cell death when co-cultured with astrocytes or microglia expressing ALS-linked C9orf72, FUS, or TDP-43. Mechanisms underlying non-cell-autonomous toxicity include loss of action potential output, altered expression levels of AMPA receptor subunits GluA1 and GluA2, and defects in neuromuscular junction (NMJ) morphology. These effects could be mediated by the extracellular secretion from glial cells of proinflammatory cytokines, such as IL-1β, IL-6, and TNFa, or TDP-43 aggregates.

Neuroinflammation, characterized by a chronic activation of microglia and astrogliosis, is a pathological hallmark of ALS pathogenesis [20]. Neuroinflammation is indeed observed at sites of motor neuron degeneration in ALS patients, both in post-mortem tissues and in vivo during disease progression using PET studies [21,22,23,24,25,26]. Accordingly, neuroinflammation has been found in rodent models of SOD1-ALS (reviewed in [27]), as well as in murine models associated with mutations in TDP-43 [28,29,30,31,32,33], FUS [34,35,36,37,38], and C9orf72 [39,40,41,42,43,44]. Although microglia and astrocyte activation are supposed to be beneficial to neuronal homeostasis, a persistent reactive state can be detrimental, and a growing amount of evidence has demonstrated that glial cells actively contribute to the onset and progression of ALS. Several studies on SOD1-ALS, mostly performed in cells and mouse models, indeed indicate that motor neuron degeneration results from the combination of motor neuron cell-autonomous mechanisms and toxicity from neighbouring non-neuronal cells, including microglia and astrocytes, in a non-cell-autonomous manner [45]. Even though data are still limited, increasing evidence suggests that a role of non-cell-autonomous toxicity might be also extended to the other ALS gene mutations, as well as to the sporadic forms of the disease (Figure 1). Indeed, although in vitro studies show that astrocytes overexpressing an ALS-linked mutant (M337V) TDP-43 or lacking TDP-43 do not affect the survival of co-cultured wild-type motor neurons [46,47], a recent study demonstrates neuronal toxicity induced by co-cultured primary astrocytes overexpressing TDP-43 [48]. This is consistent with the in vivo observation that the selective expression of M337V-TDP-43 in rat astrocytes is sufficient to induce a progressive loss of motor neurons, denervation of skeletal muscles, and consequent paralysis [31]. Moreover, different reports demonstrate that motor neurons undergo cell death when co-cultured with fibroblast-derived astrocytes from sporadic or C9orf72-ALS patients [49,50,51,52,53]. Since control motor neurons survive as a monoculture in normal media, it is conceivable that motor neuron death is mediated by a gain of toxic function mechanism rather than a lack of trophic support from astrocytes [51]. Consistently, decreased neuronal cell viability can be caused by conditioned medium from cultured mouse and human astrocytes lacking TDP-43 expression [54], expressing mutant forms of TDP-43 [55], FUS [56], and C9orf72 [57], or even overexpressing wild-type FUS [58], which is known to cause ALS pathology in patients [59,60], and wild-type TDP-43 [48]. Altogether, these results suggest that astrocytes can extracellularly secrete factors that might be toxic to neurons, including proinflammatory cytokines such as IL-1β, IL-6, and tumour necrosis factor-α (TNFα) [48,56]. A role for TNFα and its receptors (TNFR1/TNFR2), as well as for other death receptor signalling, has been already implicated in ALS pathogenesis, with major findings coming from studies in SOD1-G93A mice [61]. However, an involvement of TNFα signalling has also been observed in TDP-43 pathology [62,63,64]. Even TDP-43 aggregates can propagate from human iPSC-derived astrocytes to motor neurons in co-culture experiments [65]. Yet, the opposite direction of spreading seems to be preferential, and this process might underlie a neuroprotective action of astrocytes, since they are able to reduce mislocalized cytoplasmic TDP-43, TDP-43 aggregation, and cell toxicity in co-cultured motor neurons [65].

Mechanisms underlying non-cell-autonomous motor neuron death are still unknown. Nevertheless, it has been observed that, when co-cultured with human C9orf72 iPSC-derived astrocytes, motor neurons undergo a progressive loss of action potential output, which can be reversed by CRISPR/Cas-9 mediated excision of the repeat expansion [53]. Moreover, C9orf72 ALS frontal cortex samples are characterized by decreased transcript levels of excitatory amino acid transporters (EAAT1 and EAAT2) compared to sporadic ALS [66]. EAAT1 and EAAT2 proteins are preferentially expressed in astrocytes [67] and are responsible for glutamate uptake from synapses [68]. Thus, their reduced expression leads to excitotoxicity and progressive paralysis in rodents [68]. Interestingly, decreased EAAT1 and EAAT2 expression has also been observed in the spinal cord of transgenic rats expressing astrocytic M337V-TDP-43 [31], as well as in Drosophila overexpressing TDP-43 homologous (TBPH) in glial cells [69]. Further, conditioned medium from mouse astrocytes expressing a mutant (R521G) FUS affects the expression levels of AMPA receptor subunits GluA1 and GluA2 in cultured motor neurons, leading, also in this case, to excitotoxic damage [56]. Finally, both the expression of the wild-type form or different mutant variants of TDP-43 and the reduction of TBPH in Drosophila glial cells result in alterations in neuromuscular junction morphology, associated with defective synaptic signals and locomotor dysfunction [48,70,71]. The deletion of astroglial TDP-43 in mice also induces motor dysfunction [72].

## 3. Dysfunction of RNA/RBPs in ALS Microglia and Astrocytes

In the context of the inflammatory events that have been highlighted in models of TDP-43, FUS, and C9orf72 ALS, an obvious question is as to whether evidence exists showing that dysfunctional TDP-43 and FUS induce glia-specific alterations that might underlie those events. Similarly, whether toxic RNAs and poly-dipeptide repeat (DPR) proteins originating from C9orf72 repeat expansion have a direct impact on glia is unclear. Although much further work will be needed to resolve the issue, several observations, that will be summarized in the following paragraphs, have started to provide answers to these questions. 

### 3.1. TDP-43 and FUS

Mutations in TDP-43 and FUS have been associated with a significant fraction of both familial and sporadic ALS cases [73,74,75]. Both proteins are ubiquitously expressed DNA/RNA-binding proteins belonging to the family of heterogeneous nuclear ribonucleoproteins (hnRNPs). At a steady state, they localize predominantly in the nucleus, where they participate in different steps of gene expression regulation, including transcription, pre-mRNA splicing, and mRNA stability. However, they can shuttle continuously from nucleus to cytoplasm, where they are involved in other steps of RNA processing, including mRNA transport, translation and stress granule dynamics [76,77]. Most ALS mutations in both TDP-43 and FUS cause their re-localization from the nucleus to the cytoplasm, where they accumulate as protein aggregates. TDP-43 and FUS protein inclusions in affected neurons are indeed pathological hallmarks of ALS patients carrying respective mutations [73,78,79]. However, in 2006, wild-type TDP-43 was identified as the main component of ubiquitin-positive cytoplasmic aggregates of all cases of familial and sporadic ALS, with the notable exception of SOD1 and FUS patients, thus suggesting a more general role of TDP-43 in ALS pathology [80,81]. Pathogenic mechanisms whereby TDP-43 and FUS mutations cause ALS are still unclear. However, their subcellular mislocalization and aggregation suggest that both a loss of function due to their nuclear depletion and a gain of toxic function in the cytoplasm might contribute to ALS. According to the multiple functional roles of TDP-43 and FUS in RNA metabolism, alterations in different steps of this pathway might indeed have a primary role in motor neuron degeneration [11]. 

In addition to neuronal inclusions, glia-specific TDP-43 and FUS aggregates have been detected in ALS patients [25,73,78,79,80], thus suggesting that even both loss and gain-of-function mechanisms in microglia and astrocytes bearing mutant TDP-43 and FUS might be involved in the disease pathogenesis. Accordingly, glia-specific knockdown or overexpression of Drosophila TBPH (the fly homologue of mammalian TDP-43) are sufficient to cause, respectively, age-related motor deficits and premature lethality during larval stages [69,82]. Although the molecular mechanisms underlying TDP-43 and FUS toxicity in microglia and astrocytes are not yet defined, alterations in several cellular processes have been observed in different models in vitro and in vivo. It is known that in response to different environmental stressors both TDP-43 and FUS localize into cytoplasmic stress granules, transient membrane-less organelles where untranslated mRNAs, translational factors, and several RNA-binding proteins are stored during cell recovery, and proper dynamics of stress granules formation and dissolution is essential for cell survival. Khalfallah et al. have demonstrated that TDP-43 is critical for optimal stress granules dynamics both in mouse primary neurons and astrocytes exposed to oxidative stress [83]. Moreover, defects in glutathione induction and in the related antioxidant response following sodium arsenite treatment have been observed in primary astrocytes cultured from mutant Q331K-TDP-43 transgenic mice, as well as in M337V-TDP-43 patient fibroblasts [84]. Further, evidence for defects in astroglial metabolism has been recently provided. In particular, primary astrocytes derived from mutant transgenic mice (A315T-TDP-43) show dysfunctions in glutamate uptake and ATP accumulation [85], while the expression in rat cortical astrocytes of the C-terminal fragment of TDP-43 (208-414 aa), that forms cytoplasmic inclusions, affects lipid and glucose metabolism, thus reducing lactate release capacity, a crucial neuronal energy fuel [86]. 

Further, it has been observed that altered expression of TDP-43 and FUS in astrocytes and/or microglia is sufficient to modify their reactive states. In particular, mouse and human neural progenitor cells-derived astrocytes overexpressing wild-type FUS show increased reactivity to a proinflammatory stimulus (IL1β), compared to control astrocytes, and can induce microglia activation and neuronal cell death [58]. Similarly, the overexpression of TDP-43 in both primary microglial cells and astrocytes underlies hyperactive inflammatory response to lipopolysaccharide (LPS) or reactive oxygen species (ROS), with a higher production of proinflammatory cytokines and neurotoxic mediators compared to non-transgenic glial cells. Such response eventually increases microglia-mediated toxicity toward cultured cortical neurons, which can be reduced by inhibiting NF-kB activity [87]. Likewise, the loss of TDP-43 in primary cultured rodent microglia, but not in astrocytes, induces the expression of proinflammatory enzyme cyclooxygenase-2 (COX-2) through MAPK/ERK pathway activation, leading to the release of one of its key downstream products, prostaglandin E2 (PGE2), and eventually causing neuronal death [88]. Moreover, knockdown of TDP-43 in primary rat astrocytes causes impaired RNA homeostasis, as demonstrated by the accumulation of repetitive element transcripts and dsRNA, thus leading to a proinflammatory phenotype and to astrocyte activation [89].

Interestingly, not only endogenous levels of TDP-43 in microglia and astrocytes can modulate their activity, but it has been demonstrated that also aggregated species present in the extracellular milieu, which are found in biologic fluids of patients [90,91,92] and are considered to have prion-like properties (reviewed in [93]), can be internalized by cultured mouse microglia and induce an inflammatory response, mediated by caspase-3 activation and IL-1β and IL-18 signalling, which is cytotoxic for motor neuronal-like cultured cells [94]. Similar effects have been obtained by the exposure of mouse primary cultures of microglia to extracellular soluble forms of wild-type and mutant TDP-43 variants [64], further supporting the importance of non-cell-autonomous proinflammatory signalling in motor neuron injury caused by TDP-43.

### 3.2. C9orf72

How the hexanucleotide (G4C2) repeat expansion in the first intron of the C9orf72 gene might cause ALS neurodegeneration is currently unclear. However, both the loss of function of the protein encoded by the C9orf72 gene and a gain of function of RNA transcripts containing the expanded repeat have been to date hypothesized to mediate motor neuron degeneration [95]. 

C9orf72 protein acts in endosomal trafficking and autophagy pathway [96,97,98,99,100,101,102,103] and has a central role in maintaining immune homeostasis [104,105,106]. C9orf72 knockout mice indeed show alterations in the immune system with progressive splenomegaly, lymphadenopathy, and neuroinflammation, without showing any typical ALS neuropathological features and motor neuron degeneration [104,106]. Despite these findings support the hypothesis that gain-of-function mechanisms are primarily involved in C9orf72 disease, mouse models expressing C9orf72 repeat expansions exhibit varying degrees of neurodegeneration and do not completely recapitulate ALS pathology [40,41,107,108]. Further, haploinsufficiency in C9orf72 human motor neurons induces neurodegeneration [109]. Therefore, both loss and gain of function might contribute synergically to the disease onset and progression.

Although C9orf72 expression levels largely change across cell types and brain regions, the C9orf72 gene is highly expressed in microglia and, even if to a much lower extent, also in astrocytes [106,110], thus suggesting potentially pathological consequences of C9orf72 mutation on their proper homeostasis and functionality. 

Reduced C9orf72 transcript and protein levels have been confirmed in different brain areas of post-mortem tissues of C9orf72-ALS patients (recently reviewed in [111]), but it is still unclear whether this also occurs specifically in microglia and astrocytes. However, it has been reported that in mice, the loss of C9orf72 protein induces the upregulation of proinflammatory cytokines, altered immune responses, and lysosome accumulation in microglia, thus potentially leading to defects in the ability to remove aggregated proteins [106]. Similarly, C9orf72 knockdown in U87 glioblastoma cells or normal human astrocytes induces the formation of p62 inclusions [66], a pathological feature of C9orf72-ALS [112]. Further, these cells show altered expression of thousands of genes, including genes involved in the immune system and glutamate-associated pathways, leading to defects in glutamate metabolism [66]. 

Sense and antisense RNA foci, generated by the intracellular accumulation of expanded RNA transcripts, represent specific pathological hallmarks of C9orf72-ALS neurons [113]. Nuclear RNA foci have been also found both in microglia and in astrocytes, even if to a lesser extent compared to neurons (reviewed in [114]). The lower prevalence might be explained by several reasons, including lower RNA expression or different RNA stability. Yet, this observation is a proof of concept that G4C2 expansion might induce toxicity in these cells. In neurons, RNA foci are suggested to cause defects in RNA processing through sequestration of RNA-binding proteins [115,116,117,118,119,120,121], but it is still little explored if this might also occur in glial cells. However, a first clue comes from a report that shows a significant colocalization of the splicing factor hnRNP H with G4C2 RNA foci in cultured astrocytes from C9orf72 post-mortem tissues compared to control cells [122]. Importantly, hnRNP H sequestration has also been observed in post-mortem tissues from C9orf72-ALS patients [115,122,123], and consequent aberrant alternative splicing of multiple known hnRNP H targets has been detected in brains from both C9orf72 and sporadic ALS patients [122,124,125]. Altogether, these findings support a crucial role of hnRNP H in ALS pathogenesis, and further studies are necessary to better understand the functional relevance of the interaction between hnRNP H (as well as other RBPs) and expanded G4C2 transcripts in glial cells.

Another specific pathological hallmark of C9orf72-ALS neurons is the intracellular accumulation of five poly-dipeptide repeat (DPR) proteins (poly-GA, -GP, -GR, -PR, and -PA), unconventionally produced by repeat-associated non-AUG (RAN) translation from sense and antisense expanded transcripts [126]. In neurons, DPR toxicity seems to be mediated by the aberrant interactions of DPRs with several proteins, thus affecting their normal cellular function and leading to defects in different molecular pathways, including RNA processing and nucleus-cytoplasmic transport [127]. Despite the fact that no DPR protein inclusions are detected in glial cells both in subcortical white matter, hippocampus and white matter in the spinal cord of C9orf72-ALS post-mortem tissues [128], their expression cannot be completely excluded in these cell types. Indeed, the presence of soluble forms of DPR proteins might still compromise cell functionality, independently from their accumulation into detectable inclusions. Interestingly, depletion of the nuclear export adaptor SRSF1 in C9orf72 patient-derived astrocytes prevents motor neuron death in co-culture assays [117]. SRSF1 mediates, in concert with NXF1, the nuclear export of expanded C9orf72 RNA transcripts and its depletion are able to reduce cytoplasmic DPR expression thus preventing neurodegeneration in both in vitro and in vivo models [117]. Thus, it is conceivable that DPR expression might underlie the observed astrocyte-induced neurotoxicity. 

TDP-43-positive inclusions, which characterize C9orf72-ALS, as well as other sporadic and familial cases, have been reported in glia in different brain regions, as well as in the spinal cord of C9orf72-ALS cases [21,112,129]. These findings thus reinforce the hypothesis that alterations in RNA metabolism might occur in glial cells and contribute to ALS pathogenesis, although further investigations will be needed to understand the underlying mechanisms. 

The precise molecular cascade induced by C9orf72 mutation within microglia and astrocytes is still unknown. Yet, increasing data imply mechanisms of cell-autonomous toxicity in glial cells. It has been found that reactive microglia of C9orf72-ALS patient post-mortem motor cortex and spinal cord is characterized by a strong accumulation of LAMP1-positive lysosomes, differently from sporadic ALS samples [106], thus suggesting potential lysosomal alterations in C9orf72 cases, a pathway already strongly implicated in ALS pathology by genetic and experimental findings [130]. Altered levels of p62 and LAMP2A have been also found in microglial BV-2 cells expressing pathological repeat expansion, although functional phagocytic capability, appropriate response to an inflammatory stimulus, and normal cell viability were observed [114]. These results thus suggest that the repeat expansion does not affect microglia functional activity, but further studies in other models are needed to better understand the effect of mutation on microglial phenotypes.

Finally, both in astrocytes derived from sporadic ALS patients and in astrocytes from familial C9orf72 cases, defects in energy production have been described [131,132]. In particular, dysfunctions in adenosine, glycogen, and fructose metabolism, caused by reduced expression levels of key pathway enzymes, as well as defects in the membrane transport of mitochondrial energy substrates have been identified [131,132]. Such metabolic defects might result in insufficient nutritional support for neurons, thus potentially mediating neurotoxicity and contributing to disease progression. Indeed, circumventing adenosine metabolism dysfunction by inosine supplementation in vitro was able to improve the survival of motor neurons co-cultured with C9orf72 patient-derived astrocytes [131]. Additionally, accelerated senescence and defects in the production and/or secretion of antioxidants have been observed in human iPSC-derived astrocytes from C9orf72-ALS patients, thus leading to increased oxidative stress in astrocytes, as well as in co-cultured motor neurons, possibly contributing to neurotoxicity [49].

## 4. The Contribution of RNA Dysmetabolism to Neuroinflammation in ALS

As already mentioned, alterations in essential steps of RNA metabolism are currently believed to play key roles in motor neuron degeneration in ALS. Indeed, a wealth of experimental data demonstrated that defects in mRNA transcription and splicing, transport, and translations are associated with, and often causally involved in, the final degeneration of motor neurons (recently reviewed in [12,133]). While a specific step of RNA metabolism has not yet emerged as to be more relevant than the others, defects in mRNA splicing, transport and translation seem to play a major pathological role. Whether alterations in the latter processes are causally linked to the neuroinflammatory events that mark ALS progression is much less characterized. In the following paragraphs, we will therefore highlight the experimental evidence supporting this conclusion, as well as the circumstantial evidence that suggests that a link might indeed occur. 

### 4.1. Alternative Splicing in Reactive ALS Glia

Alternative splicing is a key process of gene expression regulation, so it is not surprising that in ALS pathological conditions that are characterized by profound changes in gene expression, alterations in underlying splicing regulation do play a role. A wealth of data has been obtained from ALS motor neurons derived from rodent models of the disease as well as from patient-derived cells, showing that defective splicing regulation occurs in ALS [36,122,125,134,135,136,137,138,139,140,141,142,143]. Much less is known about splicing dysregulation in ALS astrocytes and microglia. Early studies have provided support to the notion that alternative splicing dysregulation might also occur in astrocytes in ALS conditions. Indeed, cultured human astrocytes exposed to synthetic poly-GR and -PR C9orf72-derived dipeptides undergo significant changes in the alternative splicing of a number of mRNAs, including the mRNA encoding the glutamate transporter EAAT2, whose splicing is known to be affected in ALS patients [144,145]. This effect is possibly mediated by the interaction of C9orf72 DPRs with hnRNPA2, an RBP with known function in RNA splicing regulation, suggesting that DPR might impact on the activity of RBPs thereby affecting astrocyte functions. It is not clear, however, if these events occur in vivo in ALS astrocytes, where deposition of DPR protein inclusions are not easily detectable, as mentioned.

In genetic ALS cases due to mutations in RBPs that have a direct and established role on RNA splicing regulation, such as FUS and TDP-43, a number of potential mechanisms have been described to induce neurodegeneration. These include loss-of-function-dependent dysregulation of RNA splicing, as well as gain of splicing functions that lead to the aberrant inclusion of cryptic exons and/or introns into a range of mRNAs, including transcripts encoding RBPs [136,146], thus supporting the existence of a pathological vicious cycle that involves RBPs and that boosts splicing alterations in ALS motor neurons [147]. Similarly, dysregulation by C9orf72 repeat expansions in hnRNP H, another RBP with established roles in splicing regulation, is associated with a widespread intron retention program in human brains [141]. 

Recently, aberrant alternative splicing has been convincingly proposed to have a role in astrocyte reactivity in ALS. Valosin-containing protein (VCP), an ATPase involved in autophagosomes maturation, is mutated in 1–2% of familial ALS cases [148]. Using iPSC-derived astrocytes from ALS patients carrying the ALS-causing R155C and R191Q mutant VCP, Ziff et al. found that intron retention, which characterizes RNA transcripts from healthy astrocytes, is strikingly decreased [149]. This leads to a significant increase in the expression of the corresponding genes, which are particularly enriched into functional categories related to astrocyte reactivity regulation. Similar results were reproduced when analyzing human iPSC astrocytes derived from mutant SOD1 and C9orf72 ALS patients, as well as from astrocytes stimulated with TNFα, IL-1β, and C1q, indicating that diminished intron retention is a general feature of reactive astrocytes, including those generated by ALS pathogenic mutant genes. The mechanisms whereby VCP, SOD1, or C9orf72 mutations converge into regulation of intron retention of overlapping genes are so far unknown, but the observation from the same authors that astrocytes knocked-out for TDP-43 display a similar pattern of splicing alteration suggests that TDP-43 dysfunction, which is common to the majority of ALS cases, might have a role in the process. Importantly, these findings apparently diverge from what has been reported to occur in human motor neurons, where a significant increase in intron retention, coupled with a decreased stability and expression of the corresponding mRNAs, has been described [141,146,150,151]. The reasons for these differences are far from being clear, but they obviously indicate that the outcomes of RNA dysfunctions in ALS pathogenesis are likely cell-type specific and that therefore additional studies on RBP/RNA alterations in ALS astrocytes cells are urgently required to complete the picture of dysfunctional RNA metabolism in ALS pathogenesis. Similarly, whether and how alternative splicing alterations by dysfunctional RBPs might contribute to the detrimental effect of reactive microglia in ALS needs to be defined. Yet, it has been recently shown that misregulated alternative splicing in RBP-defective mouse microglia leads to the activation of the RhoA GTPase/ROCK pathways that, in turn, induce proinflammatory phenotypes. Importantly, these changes might be linked to several diseases of the nervous system, including neurodegenerative diseases, further supporting a possible role of these alterations in ALS [152].

### 4.2. RNA Trafficking and (Local) Translation in Reactive ALS Glia

Defective mRNA transport and translation are deeply implicated in ALS pathogenesis associated with FUS, TDP-43, and C9orf72 gene mutations. Indeed, both TDP-43 and FUS associate with proteins involved in mRNA transport, and are found in dendritic and axon terminals, supporting a role of both proteins in the regulation of mRNA transport in neurons. Moreover, they both regulate mRNA translation, either by binding to ribosomal subunits and translation factors and/or by regulating the availability of specific mRNAs for translation [76,153,154]. According to these functions, mutations in FUS and TDP-43 impact both the overall rate of translation efficiency, as well as on the synthesis of specific substrates (recently reviewed in [76]). Similarly, C9orf72 mutations elicit global protein synthesis repression by the sequestration of translation factors into G4C2 RNA foci or by interfering with translation through G4C2-derived DPRs [153,155,156,157,158]. This seems to also occur in human astrocytes, where poly-GR an -PR dipeptides induce significant changes in the expression of ribosomal rRNA genes and/or in the processing of rRNAs [144]. Moreover, it has been shown that poly-PR is able to decrease the rate of protein translation in mouse primary astrocytes [159], thus fostering the idea that C9orf72 might have a widespread impact on global translation efficiency. Notably, recent papers have revealed that ALS motor neuron pathology due to FUS mutations might be tightly linked to alterations in local translation regulation. It has been demonstrated, in fact, that ALS-linked mutant FUS accumulate into both cultured hippocampal neuron axons and sciatic nerve motor axons and that this is associated with a decreased axonal protein synthesis rate [160]. This suggests that FUS might have a role in synaptic local translation regulation, a hypothesis that is supported by the observation that in mouse and human models of FUS-ALS, FUS affects the localization and function of FMRP, another RBP with an established role in local translational regulation [161,162,163]. Importantly, glutamate receptor GLT1 expression in astrocytes and the consequent clearance of extracellular glutamate is diminished in mice knockout for FMRP [164]. This is due to a decreased expression of astroglial Gq-coupled metabotropic glutamate receptor mGluR5, whose mRNA is normally bound by FMRP and whose translation is impaired by the loss of FMRP. Although this is in contrast with the established role of FMRP as a translation inhibitor, these findings suggest the possibility, that needs to be demonstrated, that even in astrocytes an altered FMRP signalling by mutant ALS RBPs might affect local protein translation, which indeed occurs in the distal, astrocyte peri-synaptic processes [165].

## 5. Conclusions

**Figure 2:**
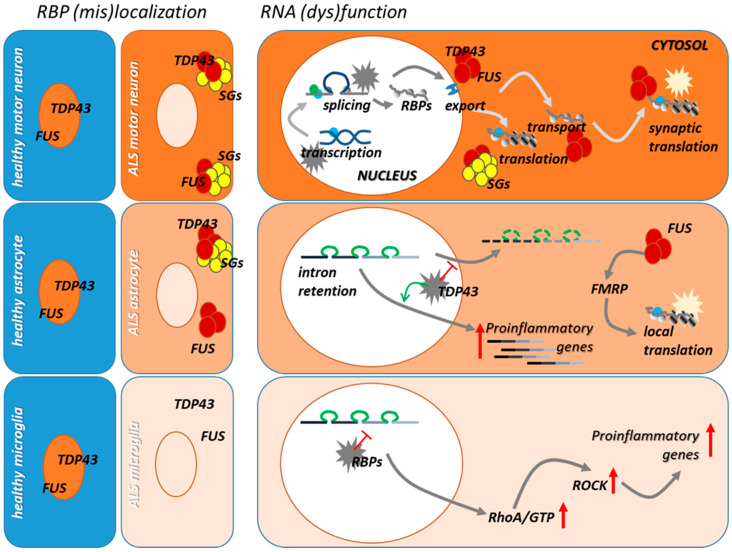
**Features of RBP/RNA dysfunction in FUS and TDP-43 motor neurons, astrocytes, and microglia.** FUS and TDP-43 are localized in the nucleus of normal cells, where they control several aspects of nuclear mRNA metabolism, including mRNA transcription and splicing. They also localize in the cytoplasm of cells where they participate in the control of mRNA transport and translation. In ALS motor neurons, FUS and TDP-43 massively delocalize in the cytoplasm, where they form cytoplasmic aggregates which frequently colocalize with stress granules (SGs). In these conditions, both gain of cytoplasmic toxic functions and loss of nuclear function contribute to the pathological mechanisms underlying motor neuron degeneration. In activated astrocytes and microglia, which are deeply involved in the progressive demise of motor neurons in ALS, cytoplasmic mislocalization of FUS and TDP-43 also occurs. In astrocytes, this leads to decreased intron retention in transcripts that regulate their reactive state, which eventually leads to an increase in their expression. Further, cytoplasmic ALS RBPs might interfere with the activity of FMRP causing alterations in local translation, which also occur in astrocyte distal processes. Dysregulation in the alternative splicing of proinflammatory transcripts also occurs in RBP-defective microglia thus supporting a role of these mechanisms in microglia activation in ALS.

**Figure 3:**
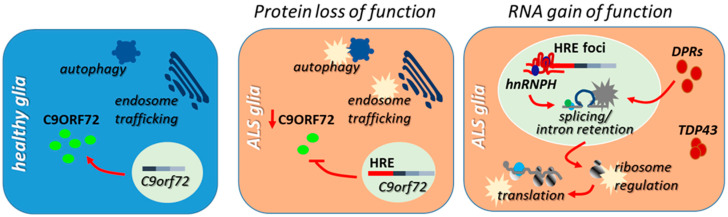
**Contribution of loss- and gain-of-function mechanisms to the inflammatory processes mediated by C9orf72-ALS astrocytes and microglia.** In ALS astrocytes and microglia, the loss of C9ORF72 protein induced by the presence of expanded hexanucleotide repeats (HRE) in the first intron of the C9orf72 gene, impacts the regulation of autophagy and endosome trafficking, and is linked to the upregulation of proinflammatory cytokines, altered immune responses, and lysosome accumulation which occur in ALS microglia and astrocytes. HRE-containing RNAs and dipeptide protein repeats (DPRs) that might accumulate in ALS glia also interfere with the regulation of splicing and translation by affecting the activities of RBPs involved in the control of RNA processing, including hnRNPH and TDP-43, and/or by interfering with ribosome biosynthesis and activity.

The concept that dysregulation of the RNA-binding protein regulatory network, which controls all the aspects of RNA processing, is deeply involved in the degeneration of motor neurons in ALS has solid grounds in an impressive set of experimental data that have been produced in the very last years. This has been mostly obtained by studying motor neurons, which indeed are the key players in ALS, but the pathological features that typify ALS-linked mutant TDP-43, FUS, and C9orf72 in neuronal cells, seem to be reproduced, at least in part, in astrocytes and microglia cells, suggesting that RNA dysmetabolism might participate to the inflammatory processes that both cell types orchestrate during disease progression (Figure 2 and Figure 3).

Certainly, a better comprehension of the mechanisms whereby dysfunctional RBPs impact the different aspects of RNA metabolism regulation, and how this contributes to the noxious effects that reactive glia exerts on motor neurons will provide further support to this conclusion and novel pathological targets to be exploited in the search of therapeutic approaches for ALS.

## Data Availability

Not applicable.
